# Development of an Integrated Bioprocess System for Bioethanol and Arabitol Production from Sugar Beet Cossettes

**DOI:** 10.17113/ftb.62.01.24.8230

**Published:** 2024-03

**Authors:** Mario Novak, Nenad Marđetko, Antonija Trontel, Mladen Pavlečić, Zora Kelemen, Lucija Perković, Vlatka Petravić Tominac, Božidar Šantek

**Affiliations:** University of Zagreb Faculty of Food Technology and Biotechnology, Department of Biochemical Engineering, Laboratory of Biochemical Engineering, Industrial Microbiology, Malting and Brewing Technology, Pierottijeva 6, 10000 Zagreb

**Keywords:** sugar beet cossettes, acid pretreatment, bioethanol, arabitol, integrated bioprocess system, biorefinery concept

## Abstract

**Research background:**

An innovative integrated bioprocess system for bioethanol production from raw sugar beet cossettes (SBC) and arabitol from remaining exhausted sugar beet cossettes (ESBC) was studied. This integrated three-stage bioprocess system is an example of the biorefinery concept to maximise the use of raw SBC for the production of high value-added products such as sugar alcohols and bioethanol.

**Experimental approach:**

The first stage of the integrated bioprocess system was simultaneous sugar extraction from SBC and its alcoholic fermentation to produce bioethanol in an integrated bioreactor system (vertical column bioreactor and stirred tank bioreactor) containing a high-density suspension of yeast *Saccharomyces cerevisiae* (30 g/L). The second stage was the pretreatment of ESBC with dilute sulfuric acid to release fermentable sugars. The resulting liquid hydrolysate of ESBC was used in the third stage as a nutrient medium for arabitol production by non-*Saccharomyces* yeasts (*Spathaspora passalidarum* CBS 10155 and *Spathaspora arborariae* CBS 11463).

**Results and conclusions:**

The obtained results show that the efficiency of bioethanol production increased with increasing temperature and prolonged residence time in the integrated bioreactor system. The maximum bioethanol production efficiency (87.22 %) was observed at a time of 60 min and a temperature of 36 °C. Further increase in residence time (above 60 min) did not result in the significant increase of bioethanol production efficiency. Weak acid hydrolysis was used for ESBC pretreatment and the highest sugar yield was reached at 200 °C and residence time of 1 min. The inhibitors of the weak acid pretreatment were produced below bioprocess inhibition threshold. The use of the obtained liqiud phase of ESBC hydrolysate for the production of arabitol in the stirred tank bioreactor under constant aeration clearly showed that *S. passalidarum* CBS 10155 with 8.48 g/L of arabitol (*Y*_P/S_=0.603 g/g and bioprocess productivity of 0.176 g/(L^.^h)) is a better arabitol producer than *Spathaspora arborariae* CBS 10155.

**Novelty and scientific contribution:**

An innovative integrated bioprocess system for the production of bioethanol and arabitol was developed based on the biorefinery concept. This three-stage bioprocess system shows great potential for maximum use of SBC as a feedstock for bioethanol and arabitol production and it could be an example of a sustainable ‘zero waste’ production system.

## INTRODUCTION

The use of renewable raw materials such as biomass from forests and agriculture could be part of the solution to the expected shortage of petroleum and other non-renewable materials and energy resources in the near future. Carbohydrate-rich renewable raw materials are usually divided into sugar-, starch- or lignocellulose-containing feedstocks (*1*-*3*). Despite competition with the food industry for raw materials containing sugar and starch, intermediate products of sugar beet processing such as thin (raw) and thick (concentrated) juice and exhausted sugar beet cossettes (ESBC; also known as a sugar beet pulp) have become popular as a raw material to produce bioethanol, particularly in geographical regions where there is overabundance of capacities to produce sugar and surplus of agricultural land (*4*, *5*). One tonne of sugar beet contains approx. 160 kg of sucrose (*w*=16 %) and refineries are able to extract approx. 130 kg of refined sugar per tonne of sugar beet (*w*>81 %). Huge amounts of ESBC, remaining as a waste from sugar production, can be considered as a valuable by-product that contains cellulose, hemicellulose, lignin and pectin (*2*, *6*-*8*) and evaluated for the production of biofuels and biochemicals using low-cost sustainable bioprocesses. The lignin content of ESBC is lower than that of grasses and softwood, which typically contain 10–20 % lignin. ESBC also contains a small amount of d-xylose (<5 % *versus* 20–35 % reported for wheat straw and corn stover) and higher amounts of l-arabinose (20–25 % in ESBC *versus* 5–15 % in grass crops) (*7*, *9*, *10*). In order to release sugars and other compounds from ESBC, numerous physicochemical pretreatment methods have been developed with different cost and energy demands, and waste stream generation (*11*, *12*). Due to the extreme conditions used, pretreatment methods usually produce inhibitory compounds that can slow down cell growth and bioprocess efficiency or inhibit enzymatic activity (*13*, *14*). Some of the already investigated methods for ESBC hydrolysis are steam explosion (*10*, *15*), liquid hot water (*16*, *17*), dilute acid pretreatment (*9*, *13*), ammonia explosion (*18*, *19*) and enzymatic digestion and depolymerization (*20*).

Monosaccharides released during ESBC hydrolysis (glucose, xylose or arabinose) have a great potential as carbon sources in various bioprocesses. For example, in conventional bioethanol production system with *S. cerevisiae*, it is possible to obtain 115 kg of ethanol from 1 tonne of enzymatically hydrolysed ESBC cellulose fraction, neglecting other fermentable sugars such as fructose, galactose, arabinose and xylose (*21*). However, novel bioprocesses require a working microorganism that can utilise different carbon sources and can adapt to different growth conditions and the presence of inhibitors. One way to improve the bioprocess is to use industrial yeasts that can easily adapt to the required conditions. Until recently, non-*Saccharomyces* yeasts were considered undesirable because they cause spoilage of food and beverages, but new research has led to the discovery of their unrecognised benefits (*22*, *23*). Some of them are interesting because they have unique properties, such as the ability to metabolise methanol, *n*-alkanes, cellulose, raffinose, arabinose, xylose, sugar alcohols and starch as carbon sources, as well as to produce large amounts of proteins and useful biochemicals (*22*, *24*). Non-*Saccharomyces* yeasts are more resistant than *Saccharomyces* yeast strains to environmental stress sources such as increased osmotic pressure, high ethanol concentrations, increased temperatures and the presence of toxic compounds. Another favourable characteristic of most non-*Saccharomyces* yeasts is their GRAS (Generally Recognized as Safe) status, which makes them desirable for handling (*22*, *25*, *26*). Sugar alcohols are interesting products of the fermentation of non-*Saccharomyces* yeasts (*27*). They are used as building block chemicals as well as low- or non-caloric sweeteners and serve as sugar substitutes in the food industry. So far, most sugar alcohols have been produced by chemical synthesis using pure sugar, but a transition towards the use of renewable, non-edible feedstocks is expected (*28*). Acid pretreatment of ESBC releases arabinose in higher concentrations than glucose or xylose and can be used for arabitol (or xylitol) production. Arabitol can be produced by chemical or microbiological processes. Chemical synthesis involves the reduction of arabinonic and lyxonic acid lactones at 100 °C using an expensive catalyst such as ruthenium or the reduction of arabinose with Na(Hg) as a catalyst. Microbiological production involves the reduction of arabinose to arabitol by aldose reductase. Additionally, the synthesis of arabitol from glucose is also possible (*19*, *29*). d-arabitol is used for the production of xylitol, ethylene glycol, propylene, arabinonic and xylonic acid, immunosuppressive glycolipids and herbicides (*30*, *31*).

High capital costs and low reaction rates are the main challenges for the setup of fermentation-based production systems in the bioeconomy, and new technologies and systems are being developed to increase the volumetric productivity of bioprocesses. In addition, the concept of restructuring the conventional systems of sugar beet industry into novel biorefinery systems to improve the energy efficiency and environmental performance of industrial sugar production systems is currently often investigated (*6*, *7*). One of the possible solutions is the integration of different technological processes (*e.g.* sugar extraction and simultaneous fermentation) to increase bioprocess efficiency and reduce energy demand (*32*, *33*). Sugar extraction on an industrial scale is carried out in vertical towers that can be described as packed bed tubular bioreactors (*34*, *35*). Tubular bioreactors (horizontal or vertical) have some advantages and disadvantages over stirred tank bioreactors. They are usually of simple construction with the possibility of different inner configurations, as well as easier to construct and scale up than stirred tank bioreactors (*36*). Mixing in tubular bioreactors is also more uniform, thus it is easier to eliminate dead zones. A packed bed tubular bioreactor is an assembly of particles that are usually of uniform size, which are randomly arranged and firmly held in position in a tube. When the fluid flows through the packed bed, a variety of physical and chemical phenomena occur in the bioreactor (*36*). In ‘contact type’ tubular bioreactors, the surface to volume ratio is significantly higher, resulting in efficient mass and heat transfer. Due to the near plug flow conditions, gradients of substrate concentration are formed along the bioreactor, which is an advantage in the case of inhibition and/or repression caused by the substrate (product), so that high productivity and optimal conversion can be achieved. This is important in bioprocesses that use solid or semisolid substrates such as raw sugar beet cossettes (SBC) (*37*, *38*). An integrated bioprocess for the production of biofuels from raw sugar beet in tubular packed bed bioreactor can be further improved by applying high cell density culture fermentation, allowing faster and more robust processes and the use of smaller reactors with increased efficiency. High biomass concentrations, obtained by retaining yeast cells or by applying high initial concentrations allow for easier cell reuse, simplified product recovery and higher dilution rates in continuous bioprocesses (*39*, *40*).

The aim of this research is to develop an innovative integrated bioprocess system for bioethanol production from raw SBC and arabitol production from the ESBC hydrolysate. The biorefinery concept was used to maximise the use of raw SBC as a feedstock for the production of the above-mentioned products.

## MATERIALS AND METHODS

### Microorganisms, raw materials and cultivation media

Non-*Saccharomyces* yeasts *Spathaspora passalidarum* CBS 10155 and *Spathaspora arborarie* CBS 11463 were purchased from CBS Fungal Diversity Centre and Technology (Utrecht, The Netherlands). Yeast *Saccharomyces cerevisiae* was obtained from the culture collection of Laboratory for Biochemical Engineering, Industrial Microbiology and Malting and Brewing Technology (Faculty of Food Technology and Biotechnology, University of Zagreb, Croatia). All yeast cultures were maintained in Petri dishes containing solid yeast extract, peptone dextrose medium (YPD), containing (in g/L): glucose 20, peptone 20, yeast extract 10 and agar 20.

The biomass of yeast *Saccharomyces cerevisiae*, which was used in the integrated bioreactor system for simultaneous sugar extraction (from raw SBC) and fermentation, was previously cultivated according to Pavlečić *et al.* (*1*, *4*). Briefly, the inoculum was prepared in Erlenmeyer flasks (*V*(medium):*V*(total)=0.4) on the raw sugar beet juice containing approx. 150 g/L of sugars with the addition of 1 g/L of (NH_4_)H_2_PO_4_. The flasks were cultivated on a rotary shaker (rotation speed of 150 min^−1^) for 18 h at 28 °C (*1*, *4*).

After cultivation of *S. cerevisiae*, the obtained biomass was separated by centrifugation (Thermo Scientific SL8R; Thermo Fisher Scientific, Waltham, MA, USA) at 8944×*g* and 4 °C for 15 min. The amount of biomass that corresponded to 30 g of dry mass was separated and washed with acid, *i.e.* yeast biomass was suspended in diluted sulfuric acid at pH=2 (500 mL) for one hour to remove possible contaminating microorganisms. The cells were then centrifuged (Thermo Scientific SL8R) for 15 min at 2830×*g* and washed 3 times with 30 mL of sterilised distilled water under aseptic conditions. The prepared *S. cerevisiae* cells were then used as inoculum in high cell density biomass experiments (*x*=30 g/L) of simultaneous sugar extraction and fermentation in the integrated bioreactor system.

Non-S*accharomyces* yeasts *Spathaspora passalidarum* CBS 10155 and *Spathaspora arborarie* CBS 11463 were cultivated on hydrolysates obtained after pretreatment of ESBC with weak acid. The inoculum preparation of the two yeasts started by transferring a loopfull of culture with standard inoculation loop (10 μL), previously grown on a solid YPD medium, into two tubes with 5 mL of liquid YPD medium. The culture was grown overnight at 28 °C and the whole amount (*i.e.* 10 mL) of the culture was transferred in Erlenmeyer flask containing 250 mL of liquid YPD medium. After further cultivation on a shaker (28 °C, 250 min^-1^, 24 h), 500 mL of cell suspension were used to inoculate the medium with ESBC hydrolysate in the stirred tank bioreactor.

The sugar beet cossettes, which were used as a raw material in simultaneous extraction and fermentation in integrated bioreactor system, were obtained by cutting in the barrel-shaped cutter Putsch® (Putsch® GmbH & Co. KG, Hagen, Germany) in Sladorana d.d., Županja, Croatia. Physical and chemical characteristics of 100 g of SBC were analyzed. The average length of a sugar beet cossette was 40.10 mm (range 5.7 to 145.5 mm), average thickness was 3.32 mm, average width was 3.57 mm, they had square-shape cross-section, while Siline number (length of 100 g of sugar beet cossettes) was 10.2 m. The soluble dry matter content of SBC varied from 145 to 185 g/L. Other parameters are listed in Table S1.

After the simultaneous extraction and fermentation, ESBC were obtained and their composition was determined according to National Renewable Energy Laboratory (NREL) procedure (*41*) (in % *m*/*m*): glucans 2.09, xylans 11.25, arabinans 11.09, formic acid 5.32, acetic acid 0.87, acid soluble lignin 23.15, insoluble lignin and ash 38.04. ESBC was first treated with weak acid in a high-pressure reactor under various conditions. A mixture of the obtained hydrolysate of ESBC with highest arabinose concentration (pretreatment conditions 180 °C, *t*_r_=5 min; 180 °C, *t*_r_=10 min; and 200 °C, *t*_r_=1 min) was used as a fermentation medium for the cultivation of yeasts *Spathaspora passalidarum* CBS 10155 and *Spathaspora arborarie* CBS 11463. The composition and concentration of the obtained ESBC hydrolysate mixture was as follows (in g/L): glucose 1.61, arabinose 14.05, xylose 2.61, formic acid 0.21 and acetic acid 2.09.

### Simultaneous extraction and fermentation of sugar beet cossettes in integrated bioreactor system

**Fig. S1:**
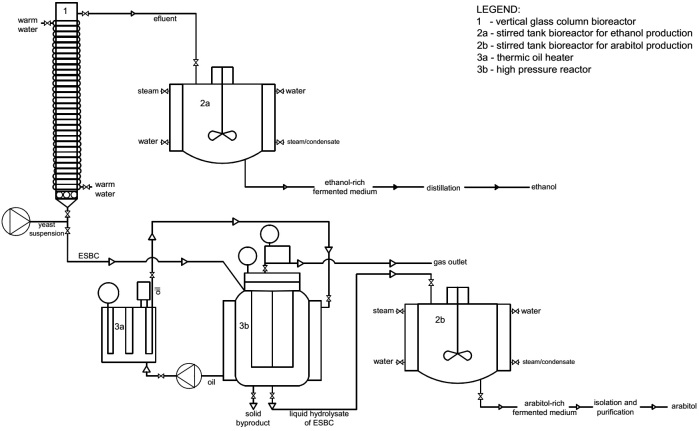
Scheme of an integrated bioprocess system for bioethanol and arabitol production from sugar beet cossettes. ESBC=exhausted sugar beet cossettes

The simultaneous extraction and fermentation of SBC was investigated in an integrated bioreactor system consisting of two connected bioreactors: a vertical packed bed column bioreactor (PBCR; height 0.5 m, diameter 5 cm, cylindrical upper and conical lower part separated by a perforated plate; Fig. S1) and a stirred tank bioreactor (STR; 2-litre vessel; B. Braun Biotech Biostat MD, Göttingen, Germany).

Vertical PBCR was filled with 0.5 kg of raw SBC and yeast suspension (30 g/L) to obtain bioreactor working volume of 1 L. The PBCR was kept at constant temperature (20, 28 and 36 °C, depending on the plan of experiments) that was maintained by a warm water flow through a spiral coil around the bioreactor. In the STR, temperature was maintained by a water jacket. Bioethanol production in the PBCR was studied in continuous mode at different combinations of medium residence time (20, 60, 120 and 180 min) and temperature (20, 28 and 36 °C). At least three working volumes of PBCR were exchanged (three residence times) before steady state conditions were established. After that, bioethanol production in the PBCR was monitored by taking samples in previously defined time intervals. The outflow of PBCR was the inflow of STR, where bioethanol was produced in repeated fed batch mode until all remaining fermentable sugars were consumed. Bioethanol was produced at pH=6.0, stirrer rotation speed of 150 min^-1^, and total and exchange volume of broth in the bioreactor of 1.5 and 1.0 L, respectively. The completion of bioethanol production in the STR was determined by refractometer (Atago N-20; Atago Co., Ltd, Tokyo, Japan) and additionally confirmed by chromatographic analysis of fermentable sugars in the broth. Bioprocess in the STR was also monitored by taking broth samples. In the bioreactor system, pH and pO_2_ were monitored by appropriate probes. Bioethanol production in the integrated bioreactor system was examined without medium recirculation.

### Weak acid pretreatment of ESBC in the high-pressure reactor

A high-pressure reactor (HPR) was used for weak acid pretreatment of ESBC. The HPR is constructed as a double jacket vessel (total volume of 20 L) without stirrer and perforated holding vessel (slightly smaller diameter than the reactor) for ground feedstock. The pretreatment of the ESBC was carried out according to Marđetko *et al.* (*11*). A mass of 1 kg of ESBC (containing *w*(dry mass)=92 %) was transferred into high-pressure reactor, suspended in 10 L of diluted sulfuric acid (*w*=0.5 %) and mixed until homogenous suspension was obtained by an external stirrer. The ESBC suspension was used in weak acid pretreatment experiments at various temperatures (160, 180 and 200 °C) and residence times (*t*_r_) of 1, 5 and 10 min. The obtained ESBC hydrolysate was neutralized with Ca(OH)_2_, left to sediment and then the liquid part of the hydrolysate was decanted. Solid phase of the ESBC hydrolysate was then rinsed with warm distilled water until the neutral pH value of the water was achieved at the outlet of the filtration unit. Both liquid and solid phase were stored, analysed for their composition and kept at -20 °C to investigate the cultivation of *Spathaspora passalidarum* CBS 10155 and *Spathaspora arborarie* CBS 11463.

### Production of arabitol from ESBC hydrolysate by using non-Saccharomyces yeasts

A volume of 4.6 L of the produced liquid phase of ESBC hydrolysate supplemented with yeast extract (10 g/L) and peptone (20 g/L) was used as a nutrient medium for cultivation of yeasts *Spathaspora passalidarum* CBS 10155 and *Spathaspora arborarie* CBS 11463 in the STR (Sartorius Biostat Cplus, Göttingen, Germany). After *in situ* sterilization in the bioreactor (121 °C for 20 min) and cooling, the pH value was automatically adjusted in the STR using 2 M NaOH solution. Cultivation lasted 48 h at 28 °C and pH=6. The air flow was 2.5 L/min and the stirrer speed was 550 min^-1^. The pH value of the substrate during the bioprocess was regulated by adding 2 M H_2_SO_4_ or 2 M NaOH.

### Analytical methods and calculation of bioprocess efficiency parameters

Biomass concentration was determined either by measuring the absorbance or by determining the dry matter of the biomass (*11*). The composition of carbohydrates, alcohols and organic acids in the SBC fermentation samples and weak acid ESBC hydrolysates were determined by ultra pressure liquid chromatography coupled with a refractive index detector (UPLC RID) analysis according to Marđetko *et al.* (*11*). Bioprocess efficiency parameters were calculated according to Pavlečić *et al.* (*42*):


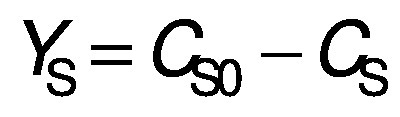
 /1/


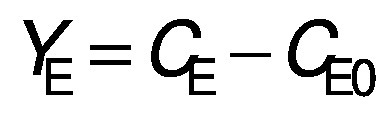
 /2/


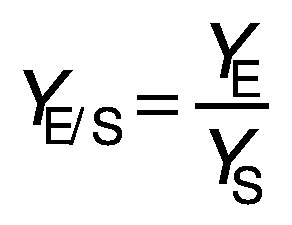
 /3/


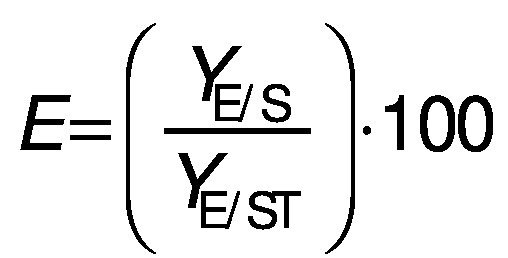
 /4/


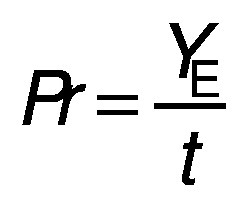
 /5/

where *Y*_S_ is total consumption of substrates (g/L), *C*_S_0__ is initial concentration of substrate (g/L), *C*_S_ is final concentration of substrate (g/L), *Y*_E_ is total ethanol yield (g/L), *C*_E_ is final concentration of ethanol (g/L), *C*_E_0__ is initial concentration of ethanol (g/L),*Y*_E/S_ is conversion coefficient of substrate to ethanol (g/g), *E* is bioprocess efficiency (%), *Y*_E/ST_ is theoretical conversion coefficient of substrate into ethanol (g/g; 0.538 g/g) *Pr* is bioprocess productivity (g/(L^.^h) and *t* is time (h).

Theoretical substrate conversion into ethanol (*Y*_E/ST_) was 0.538 g/g because the main sugar in the raw SBC is sucrose (*42*). In this study, all experiments were repeated at least once and the standard deviation of all measurements was in the range of experimental error (below 4.7 %).

## RESULTS AND DISCISSION

### Simultaneous extraction and fermentation process in the integrated bioreactor system at various temperatures and residence times

Simultaneous extraction and fermentation of raw sugar beet cossettes (SBC) was carried out in the integrated bioreactor system (consisting of vertical (PBCR) and stirred tank bioreactor (STR); Fig. S1) where high density suspension of yeast *Saccharomyces cerevisiae* (30 g/L) was used as extracting agent instead of tap water. The sugar extracted in the PBCR was simultaneously fermented into ethanol by the high-density yeast culture in continuous mode. This bioprocess was investigated with different combinations of medium residence time (20, 60, 120 and 180 min) and temperature (20, 28 and 36 °C) as the most important parameters for bioethanol production in the PBCR. However, the complete conversion of fermentable sugars into ethanol was achieved in the STR as the second stage of integrated bioreactor system. In the second stage (STR), bioethanol was produced in repeated fed batch mode to achieve the highest efficiency of bioethanol production. In all experiments, sucrose was not detected in the PBCR outflow, indicating rapid hydrolysis of sucrose by invertase to glucose and fructose as a consequence of high yeast cell density. The idea of using such a high density of yeast cells was based on the known facts regarding high cell density fermentation (*14*, *15*) to increase ethanol production rate. The use of high cell density cultures can provide faster bioprocesses using smaller bioreactors such as PBCR with higher volumetric ethanol productivity (*e.g.* 0.503 g/(L^.^h)) for STR (*4*) than 6 g/(L^.^h) for PBCR (*28*, *43*). Furthermore, in such bioprocesses it is easier to remove and reuse the cells, which simplifies the recovery of the product (*39*, *40*). Intermediates of sugar beet processing, *e.g.* raw SBC, can be successfully used in various bioprocesses as reported in the literature (*1*-*4*, *43*). It is well known that *S. cerevisiae,* depending on the oxygen concentration in the cultivation medium, changes metabolism from aerobic, microaerobic to anaerobic metabolism. Among yeasts, *S. cerevisiae* and other *Saccharomyces* species are unique because they are able to restrict aerobic and increase anaerobic glucose metabolism even in the presence of oxygen by suppressing respiratory genes. Under these conditions, *S. cerevisiae* simultaneously oxidises ethanol and degrades glucose aerobically to produce energy and ingredients for its basal metabolism and biomass growth (*44*).

**Fig. 1:**
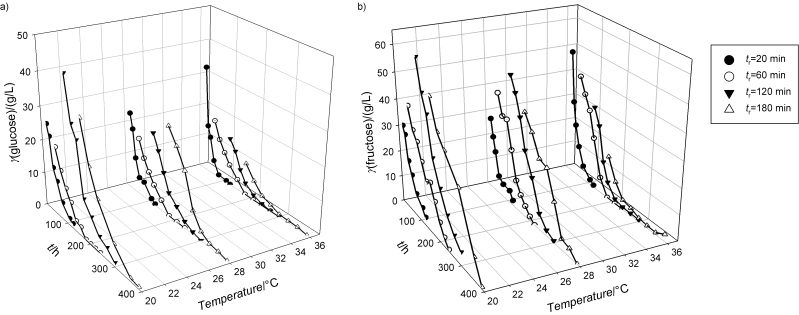
Concentrations of: a) glucose and b) fructose during simultaneous extraction and fermentation of raw sugar beet cossettes (SBC). Bioprocess was performed by continuous extraction of SBC using yeast suspension (*x*=30 g/L) at three different temperatures and four different medium residence times in the integrated bioreactor system

**Fig. 2:**
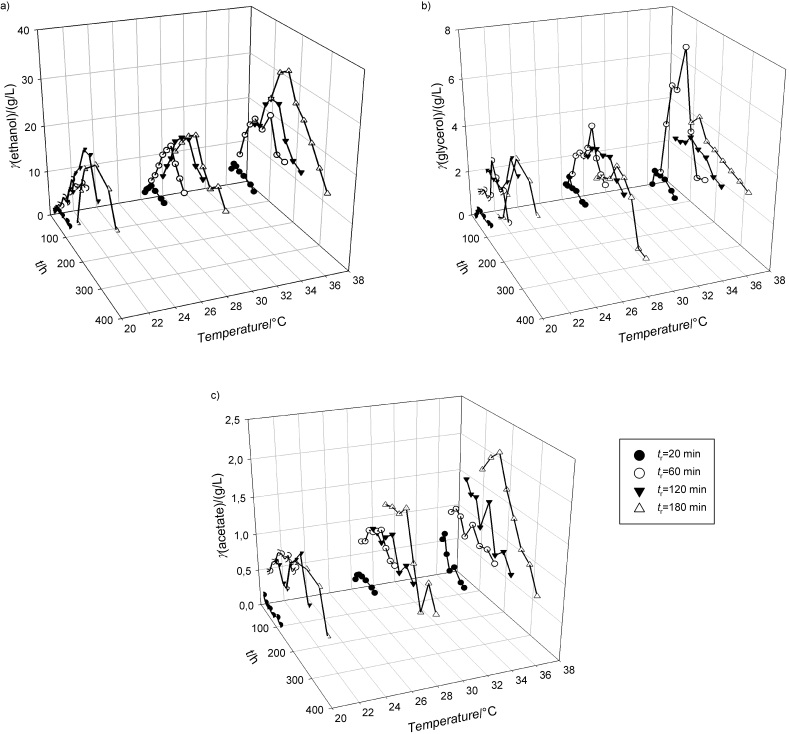
Concentrations of: a) ethanol, b) glycerol and c) acetate during simultaneous extraction and fermentation of raw sugar beet cossettes (SBC). Bioprocess was performed by continuous extraction of SBC using yeast suspension (*x*=30 g/L) at three different temperatures and four different medium residence times in the integrated bioreactor system

In our study, bioethanol was produced under microaerobic conditions, which can be confirmed by the pO_2_ measurement (in the range of 1−2 % air saturation of the medium determined by oxygen probe) during this study. The flow of the medium through the bioreactor system supports its mixing and thus the oxygen dissolution in the cultivation broth. In addition, different yeast fermentation products (*e.g.* ethanol, acetate, glycerol or biomass) were observed during this study, which is additional confirmation of microaerobic conditions in the bioreactor system (Fig. 1 and Fig. 2). It has to be pointed out that the yield of yeast biomass under anaerobic conditions is lower than under aerobic conditions. Acetate is not produced under anaerobic conditions, which is consistent with the results of Blomqvist *et al.* (*45*). The decrease in ethanol and glycerol concentrations and the increase in acetate and biomass concentrations correlate with the increase in dissolved oxygen concentration in the cultivation broth. The coupling effect between ethanol production and yeast growth can be explained by the energy balance with the following main metabolic reactions: oxidative phosphorylation, production of ethanol, glycerol, acetic acid, succinic acid and theoretical yields of production/ATP consumption (*46*, *47*).

As shown in Fig. 1, sugar extraction from SBC and bioethanol production efficiency depended on temperature and residence time of the medium (*1*, *48*). Three temperatures were tested: 20 °C (room temperature), 28 °C (the optimal growth temperature of *S. cerevisiae*) and 36 °C (the maximum tolerable growth temperature of *S. cerevisiae*) (*49*). One of the objectives of the conducted studies in the bioreactor system was to determine the relationship between bioethanol production and residence time during the simultaneous extraction and fermentation of SBC. Therefore, four residence times (*t*_r_=20, 60, 120 and 180 min) were investigated.

At a residence time of 20 min, the sugar content (glucose and fructose) in the liquid phase of the medium was higher at 36 °C than at 20 or 28 °C, as the sugar extraction efficiency increased with temperature (Fig. 1a and Fig. 1b). Maximum ethanol concentrations observed in the bioreactor system at each temperature increased with the increase of temperature (Fig. 2a). Glycerol and acetate concentrations also increased with temperature (Fig. 2b and Fig. 2c), although some fluctuations in their concentrations were observed. These fluctuations in the concentration of different yeast metabolism products during the bioprocess were a consequence of theheterogenity of the system (dilution effect due to plug flow conditions and yeast distribution heterogenity through the raw SBC layer in the bioreactor system (*1*, *28*)) and analytical errors.

The efficiency of sugar extraction also depends on the residence time (*t*_r_) and consequently longer residence times must be favourable for the extraction/fermentation process. The increase in residence time was expected to be related to the higher ethanol concentrations in the extraction/fermentation bioprocess. Therefore, the residence time of the medium in the bioreactor system was increased at *t*_r_=60 min (Fig. 2a and Fig. 2b) and consequently higher glucose and fructose concentrations were observed than in experiments with *t*_r_=20 min. Furthermore, higher maximum ethanol concentrations (28.31 g/L at 36 °C) were observed (26.80 g/L at 20 °C and 23.18 g/L at 28 °C) due to the prolonged time for sugar extraction and conversion to ethanol, glycerol and acetate (Fig. 2a, Fig. 2b and Fig. 2c). Based on the obtained results, it is clear that the bioprocess behaviour at *t*_r_=60 min was similar to the bioprocess behaviour at *t*_r_=20 min.

Further increase of residence time to 120 min in the bioreactor system resulted in the highest concentrations of sugars (glucose and fructose; Fig. 1a and Fig. 1b) in the bioreactor system (even at lower temperatures) compared to all experiments performed in this study. These results clearly showed that optimal conditions for sugar extraction in the integrated bioreactor system were at 20 °C and *t*_r_=120 min. Based on previous findings, it is obvious that residence time had more pronounced effect on the sugar extraction than the examined temperature range. Increased efficiency of sugar extraction was related to the highest ethanol concentrations in the outflow of the bioreactor system, which were 32.83, 29.24 and 30.71 g/L at 20, 28 and 36 °C, respectively. In Fig. 2a, it can be seen that maximum ethanol concentrations were detected after 270 min at 20 °C, 240 min at 28 °C, and 210 min at 36 °C. Under these conditions, glycerol and acetate concentration profiles (Fig. 2b and Fig. 2c) followed the same pattern as ethanol concentration profiles, which is in agreement with literature (*46*, *47*). At the end of bioprocess, a decrease in the concentration of ethanol and other metabolites (gycerol and acetate) was observed. At residence time of 120 min, the highest decrease in ethanol concentration (compared to the maximum concentration) was detected at 20 °C (25.22 %) and followed by the decrease at 28 °C (15.01 %) and at 36 °C (14.69 %). This phenomenon was observed at glucose concentrations below 5 g/L and it can be explained as a consequence of ethanol utilization by increased yeast concentration (30 g/L) under the semiaerobic conditions for cell maintanance and growth (*46*, *47*). Furthermore, the heterogenity of the bioreactor system and the resulting analytical errors are additional effects that contribute to the above-mentioned phenomenon. The same discussion can explain the decrease of gycerol and acetate concentrations (Fig. 2b and Fig. 2c).

Somewhat higher maximum ethanol concentrations were observed in the bioreactor system at 28 °C (29.22 g/L) and 36 °C (35.98 g/L) when the medium residence time was increased to 180 min (Fig. 2a). Maximum ethanol concentrations in the bioreactor system were reached after 300 min at 20 °C, 270 min at 28 °C and 240 min at 36 °C. At the same time, glycerol and acetate concentrations at all temperatures were approximately similar to those observed at residence time of 120 min (Fig. 2b and Fig. 2c). The decrease of ethanol concentration (compared to the maximum concentration) at residence time of 180 min can also be seen when glucose concentration was below 5 g/L. The highest decrease in ethanol concentration (compared to the maximum concentration) was observed at 36 °C (31.91 %), followed by a decrease at 28 °C (23.10 %) and 20 °C (21.73 %). This phenomenon was already observed at the residence time of 120 min, where ethanol concentration decreased due to yeast metabolism under semiaerobic conditions as previously stated (*46*, *47*).

Based on the results obtained during the simultaneous extraction and fermentation process, efficiency parameters were calculated and shown in Table 1. The summarized efficiency parameters of integrated bioprocess system are total mass of extracted sugars and concentrations (the outflow of PBCR and STR) for glucose, fructose and ethanol as well as bioethanol production efficiency. Total mass of sugars that can be extracted (independent of temperature and residence time) from 500 g of raw SBC was in the range of 46.00-56.42 g and it depends on the properties of the raw material used in the experiments. The maximum bioethanol production efficiency in the bioreactor system (87.22 %) was achieved at 36 °C and residence time of 60 min. Further increase of residence time (at 120 and 180 min) resulted in similar efficiency of bioethanol production (79.69−87.10 %). These results show that the prolongation of residence time over 60 min did not improve bioprocess efficiency, but reduced the bioprocess volumetric productivity. Under these conditions, the effect of temperature on the bioprocess efficiency was not significant although some small improvements were observed (Table 1). Ethanol production in the PBCR, compared to total bioethanol production of the integrated bioreactor system, increased with higher temperatures and longer residence times. Some fluctuations were observed at lower residence times (20 and 60 min) as a consequence of SBC heterogeneity in the bioreactor system and analytical errors. The PBCR ethanol outflow in the integrated bioreactor system was in the range 49.44−92.12 % of the total bioethanol production (Table 1). These results clearly show that the second stage of bioreactor system (STR) is required for complete sugar conversion into ethanol and consequently to achieve the highest bioprocess efficiency. It has to be pointed out that bioethanol production from SBC can be completely done in the PBCR, but bioreactor construction and operational conditions need to be further optimized. This will be the aim of our further research related to the bioethanol production in the PBCR.

**Table 1:** Extraction and bioethanol production efficiency in the integrated bioreactor system with sugar beet cossettes (SBC) as a feedstock

*t*(residence)/min		20			60			120			180		
Temperature/°C		20	28	36	20	28	36	20	28	36	20	28	36
*m*(extracted sugar)/g*		46			53.42			56.42			53.7		

PBCR outflow *γ*/(g/L)	Glucose	11.40	7.96	9.20	8.99	16.32	10.34	37.96	12.12	9.94	15.40	14.22	6.51
	Fructose	17.20	13.53	21.46	22.60	33.14	38.58	57.46	40.31	24.23	25.15	28.33	8.15
	Ethanol	4.52	6.60	8.10	8.49	14.25	16.06	12.54	16.24	22.58	12.38	19.11	22.57

STR outflow *γ*/(g/L)	Glucose	0.72	0.34	0.08	0.07	0.14	0.10	0.87	0.95	0.00	0.59	0.70	0.00
	Fructose	2.15	0.44	0.80	0.32	1.10	0.34	2.62	1.02	0.51	2.52	3.22	1.10
	Ethanol	6.87	7.89	10.58	17.17	20.80	24.86	24.55	24.85	26.20	21.69	22.47	24.50
Ethanol production efficiency/%		29.61	32.43	43.58	60.18	74.09	87.22	86.21	84.83	87.10	79.69	83.90	86.57
*w*(PBCR in total ethanol production)/%		65.79	83.65	76.56	49.44	68.51	64.60	51.08	65.35	86.18	57.08	85.05	92.12

*Total mass of fermentable sugars that can be extracted from 500 g of raw SBC. STR=stirred tank bioreactor, PBCR=packed bed column bioreactor

### Weak acid pretreatment of ESBC in the high-pressure reactor under various conditions

Agricultural waste, such as ESBC, can be used for the production of biofuels and biochemicals. After appropriate pretreatment, lignocellulosic materials can release monosaccharides which can then be converted into various products by fermentative and biocatalytic routes (*1*, *7*, *9*). Several studies have shown the effectiveness of weak acid pretreatment (*9*, *11*, *13*) and therefore such an approach was used in our research. The tested temperatures and residence times were selected according to Marđetko *et al.* (*11*) and the obtained results are shown in Table 2.

**Table 2:** Concentrations of glucose, xylose, arabinose, acetic acid, and total furans in the exhausted sugar beet cossettes (ESBC) hydrolysate after weak acid pretreatment

**Temperature** **/°C**	***t*_r_/min**		***γ*/(g/L)**				
Glucose	Xylose	Arabinose	Acetic acid	Total furans
**160**	1	0.13		1.60	7.10	0.11	0.02
	5	0.34		1.68	9.61	0.13	0.01
	10	0.16		1.53	8.35	0.13	0.02
**180**	1	0.24		1.36	8.63	0.11	0.00
	5	0.91		1.53	10.64	0.15	0.00
	10	1.21		1.82	10.93	0.17	0.00
**200**	1	0.31		2.64	12.30	0.13	0.00
	5	0.22		1.87	9.43	0.16	0.00
	10	0.29		0.91	2.19	0.03	0.00

After weak acid pretreatment of ESBC in a high-pressure reactor at 160 °C, the highest yield of arabinose and glucose was found in the hydrolysate obtained at retention time of 5 min (9.61 g/L of arabinose and 0.34 g/L of glucose in 3.5 L of liquid phase). The highest yield of xylose was recorded at a retention time of 1 min and the same temperature: 1.60 g/L of xylose in 3.8 L of the liquid ESBC hydrolysate phase. The highest yields of carbohydrates at 180 °C were obtained at retention times of 10 min for arabinose (11.36 g/L of arabinose in 3.4 L of liquid hydrolysate) and 5 min for xylose and glucose (1.70 g/L of xylose and 0.30 g of glucose in 3.63 L of liquid hydrolysate). Pretreatment of the ESBC at 200 °C resulted in the highest concentrations of fermentable sugars at a retention time of 1 min (12.22 g/L of arabinose, 2.64 g/L of xylose and 0.31 g/L of glucose in 3.6 L of the liquid phase of the hydrolysate). At longer retention times (5 and 10 min), there was a significant decrease in the amount of soluble arabinose, which indicates its relatively high thermal instability. According to Kühnel *et al.* (*9*), more product is lost at high temperatures due to the formation of volatile compounds and Maillard-type reactions. The high rate of arabinose release from ESBC at temperatures from 150 to 175 °C has already been confirmed in earlier research (*13*, *14*, *50*). In addition to an increase in the solubility of monosaccharides in the liquid phase of the hydrolysate, significant losses in the mass of the sample occur, and such results were also confirmed in our research. A clear change in the substrate colour to dark brown and a specific caramel aroma were observed after pretreatment at higher temperatures and longer residence time (200 and 180 °C/10 min), which is in agreement with Cadete *et al.* (*51*). In addition to released fermentable sugars, undesired products can also be formed or released in the acidic pretreatment and subsequently negatively affect fermentation. Acetic acid, furfural, 5-hydroxymethylfurfural (HMF) and formic acid are the compounds that were detected after acid pretreatment. The highest yield of acetic acid was observed at 200 °C with a retention time of 5 min (0.165 g/L in 3.4 L of liquid hydrolysate) and at 180 °C with retention time of 10 min (0.17 g/L in 3.6 L of liquid phase of ESBC hydrolysate). The yields of acetic acid were significantly higher than those of furan, whose highest concentrations were recorded at 160 °C and 10 min retention time (0.017 g/L in 3.6 L of liquid ESBC hydrolysate). At 180 and 200 °C furans were not determined as their concentrations were below minimum detection level (>0.01 g/L) of the UPLC system.

### Cultivation of yeasts Spathaspora passalidarum CBS 10155 and Spathaspora arborarie CBS 11463 in the hydrolysed ESBC medium

**Fig. 3:**
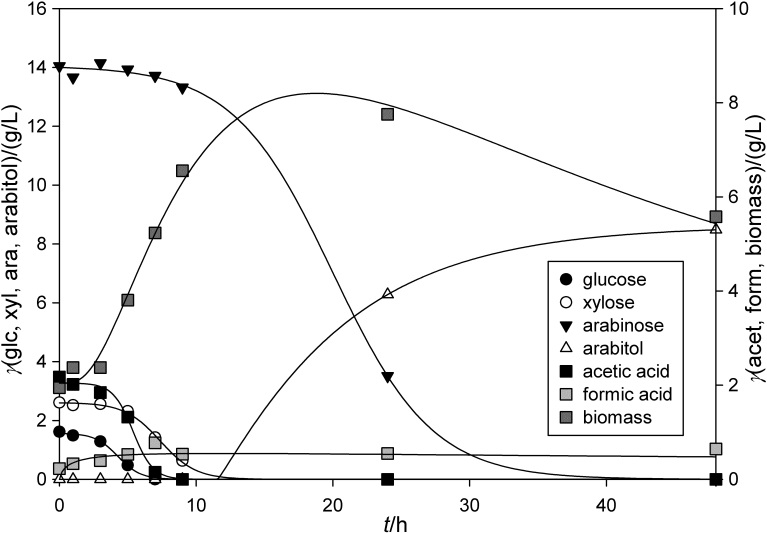
Concentration profiles during cultivation of *Spathaspora passalidarum* CBS 10155 in the stirred tank bioreactor (STR) on the weak acid exhausted sugar beet cossette (ESBC) hydrolysate

Monosaccharides from lignocellulosic waste materials, *e.g.* xylose and arabinose from ESBC, represent valuable carbon sources, especially for non-*Saccharomyces* yeast from genus *Spathaspora* for the production of different biochemicals such as sugar alcohols (*24*, *29*, *51*). In this study, the yeasts *Spathaspora passalidarum* CBS 10155 and *Spathaspora arborarie* CBS 11463 were cultivated on weak acid ESBC hydrolysate. The nutrient medium was obtained by mixing three ESBC hydrolysates from a high-pressure reactor. At the beginning of cultivation, the composition of the nutrient medium (in g/L) was arabinose 14.05, xylose 2.61, glucose 1.62, acetic acid 2.10 and formic acid 0.21 (Fig. 3). The concentrations of acetic and formic acids were below the reported inhibitory concentrations of 2.5 and 1 g/L, respectively (*52*).

During cultivation, the yeast *Spathaspora passalidarum* CBS 10155 consumed glucose, xylose and arabinose as carbon sources (Fig. 3). Glucose was completely consumed in the 6th hour of cultivation, xylose in the 24th hour and arabinose was completely consumed after 48 h, which shows typical sequential utilization of substrates already determined by *Saccharomyces* and non-*Saccharomyces* yeasts (*51*, *52*). The maximum consumption rate of glucose was 0.496 g/(g^.^h), while for arabinose it was only 0.086 g/(g^.^h), calculated according to Pavlečić *et al.* (*42*). The yeast entered the exponential phase of growth in the 4th hour of cultivation and the stationary phase after the 10th hour. The synthesis of arabitol began after entering the stationary growth phase. After 24 h of cultivation, the concentration of arabitol was 6.28 g/L. The maximum concentration of 8.48 g/L was reached in the last hour of cultivation. Ethanol was not detected during this cultivation. The concentration of acetic acid decreased by 93 % before and during the exponential growth phase (0.151 g/L), and after the 8th hour of cultivation it was not detected in the medium. The conversion coefficient of the substrate (arabinose) into arabitol was *Y*_P/S_=0.603 g/g with the productivity of 0.176 g/(L^.^h), which is in accordance with literature (*19*, *31*). In comparison, Dien *et al*. (*53*) achieved the yield of 0.73 g/g of arabitol produced from sugar beet arabinose using *S. passalidarum*. Glaser *et al.* (*54*) estimated that it is possible to produce 129.6 kg of arabitol from 1 t of pretreated sugar beets (containing 189 kg of glucose and 177 kg of arabinose) using non-*Saccharomyces* yeasts (*Y*_P/S_=0.7 g/g). Higher arabitol yields (*Y*_P/S_=0.9 g/g) can be achieved by using *Meyerozyma caribbica,* a pentose-fermenting yeast (*55*).

**Fig. 4:**
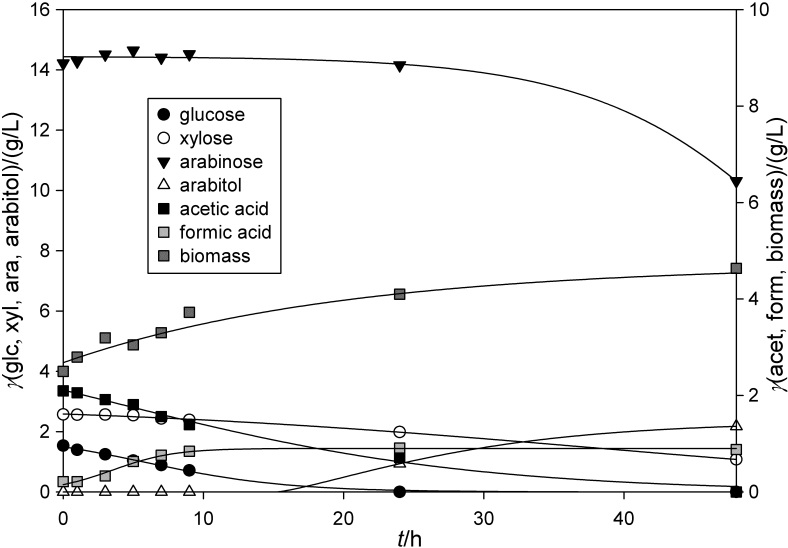
Concentration profiles during cultivation of yeast *Spathaspora arborarie* CBS 11463 in the stirred tank bioreactor (STR) on the weak acid exhausted sugar beet cossette (ESBC) hydrolysate

During the cultivation of *Spathaspora arborarie* CBS 11463, of the three carbohydrates present in the medium, only glucose was completely consumed (Fig. 4). The initial xylose concentration decreased by 58 % (1.077 g/L). The maximum consumption rate of glucose was 0.079 g/(g^.^h) and of arabinose 0.016 g/(g^.^h). At the end of cultivation, 10.313 g/L of arabinose remained in the medium, which means that only 27.5 % of the initial concentration of arabinose was consumed. Corresponding to the lower growth and consumption rate of arabinose, less arabitol was produced. At the end of cultivation, the concentration of arabitol was 2.175 g/L, which was 3.9 times less than the concentration of arabitol produced by the yeast *Spathaspora passalidarum* CBS 10155 on the same substrate. The conversion coefficient of arabinose into arabitol was *Y*_P/S_=0.557 g/g, and the arabitol synthesis productivity was 0.045 g/(L^.^h). The obtained bioprocess efficiency data were within the range of the literature (*19*, *31*). The concentration of acetic acid in the substrate decreased linearly until the 24th hour of cultivation and it was not detected at the end of cultivation.

## CONCLUSIONS

The obtained data obviously showed that the simultaneous extraction and fermentation in the integrated bioreactor system depended on the operational parameters of the bioprocess (residence time and temperature). The efficiency of bioethanol production was improved with the increase of both bioprocess operational parameters. The highest bioethanol production efficiency of 87.22 % was observed at residence time of 60 min and temperature of 36 °C. Further increase in residence time did not result in considerable improvement of bioethanol production efficiency. The largest part of bioethanol was produced in the packed bed column bioreactor (PBCR; 49.44–92.12 %) and residual part in the stirred tank bioreactor (STR; second stage of integrated bioreactor system), which was used for complete conversion of fermentable sugars into bioethanol. Results of exhausted sugar beet cossettes (ESBC) hydrolysis showed that the highest sugar yield was reached at 200 °C and residence time of 1 min with following sugars concentrations in the liquid phase of ESBC hydrolysate (in g/L): glucose 0.297, arabinose 11.892 and xylose 2.573. In addition, the concentration of weak acid pretreatment inhibitors was below inhibitory levels (*e.g.* 2.5 g/L acetic acid and 1 g/L formic acid). The use of liqiud phase of ESBC hydrolysate for arabitol production by *Spathaspora passalidarum* CBS 10155 showed that 8.48 g/L of arabitol can be produced with the conversion coefficient of arabinose into arabitol of *Y*_P/S_=0.603 g/g and bioprocess productivity of 0.176 g/(L^.^h). Based on the obtained results, it is clear that further optimization of integrated bioprocess system for bioethanol and arabitol production from sugar beet is needed. The development of integrated bioprocess system using sugar beet as a substrate for bioethanol and arabitol production has considerable potential for industrial application. Other lignocellulose-containing raw materials could also be used as substrates in this type of integrated bioprocess system.

## Appendix

**Table S1:** Physical and chemical properties of sugar beet cossettes

Parameter		Value
*N*(sugar beet cossette)		273
*N*(short cossette, *l*<10 mm)		19
*N*(standard cossette, 10 mm<*l*≤50 mm)		200
*N*(long cossette, *l*>50 mm)		54
*A*_total_/m^2^		0.140929
*V*_total_/m^3^		0.000123
Silin number/m		10.2034
*A*_specific_=(*A*_total_/*V*_total_)/(1/m)		1145.421
*m*(total dry matter)/*m*(cossette)		0.22±0.03
*m*(soluble dry matter)/*m*(cossette)		0.16±0.03
*m*(sucrose)/*m*(dry matter)		0.900±0.006
*m*(glucose)/*m*(soluble dry matter)		0.021±0.003
*m*(fructose)/*m*(soluble dry matter)		0.024±0.003
*m*(non-soluble dry matter)/*m*(cossette)		0.055±0.001
*m*(non-soluble dry matter)/*m*(total dry matter)		0.26±0.03
